# TNM cancer staging: can it help develop a novel staging system for type 2 diabetes?

**DOI:** 10.2147/DMSO.S179963

**Published:** 2018-11-28

**Authors:** Moahad S Dar, Sami A Bég

**Affiliations:** 1 Department of Veteran Affairs, Greenville Health Care Center, Greenville, NC, USA, moahad.dar@va.gov; 2 Division of Endocrinology & Metabolism, Department of Internal Medicine, Brody School of Medicine at East Carolina University, Greenville, NC, USA, moahad.dar@va.gov; 3 Proactive Living Inc., Columbia, SC, USA

**Keywords:** type 2 diabetes, macrovascular complications, microvascular complications, hemoglobin A1C, glomerular filtration rate, GFR, TNM cancer staging

## Abstract

Type 2 diabetes (DM2) constitutes 90%–95% of the diabetes cases and is increasing at an alarming rate in the world. The Centers for Disease Control and Prevention (CDC) estimates that more than 29 million people in the United States have diabetes, which often causes mortality from macrovascular complications and morbidity from microvascular complications. Despite these troubling facts, there is currently no widely accepted staging system for DM2 like there is for cancer. TNM oncologic staging has taken a complex condition like cancer and conveyed likelihood of survival in simple alpha-numeric terms that both patients and providers can understand. Oncology is now entering the era of precision medicine where cancer treatment is increasingly being tailored to each patient’s cancer. In contrast, DM2 lacks a staging system and remains a largely invisible disease even though it kills more Americans and costs more to treat than cancer. Is a comparable staging system for DM2 possible? We propose the Diabetes Staging System for DM2 that utilizes macrovascular events, microvascular complications, estimated glomerular filtration rate (GFR), and hemoglobin A1C to stage DM2.

## Background

Type 2 diabetes (DM2) constitutes 90%–95% of the diabetes cases and is increasing at an alarming rate in the world with 425 million people affected in 2017 and 629 million projected in 2045. The Centers for Disease Control and Prevention (CDC) estimates that 29 million people in the US have DM2, which costs the US economy $322 billion.^1^ More concerning is that DM2 cases are expected to rise sharply over the next 40 years due to an aging population, larger numbers of minority groups at higher risk to develop DM2, and longer life expectancy.^2^ Effective strategies are urgently needed to ease this burden on patients, the health care system, and the nation.

Disease staging systems are one approach that can help. The main objective of any good staging system is to indicate prognosis, facilitate information exchange, assist providers in planning and evaluating treatment, and aid research efforts.^3^ The field of oncology has successfully used cancer staging systems, such as TNM, Ann Arbor, FIGO, and Dukes, to accomplish these objectives and is now entering the era of precision medicine. A tangible example is a Phase II clinical drug trial of melanoma that supported “the rationale for the analysis of blood and tumor samples in future studies to expedite the development of more effective treatments”.^4^ In contrast, DM2 lacks a coherent staging system and physicians are not currently able to tailor treatment to each patient. Thus, DM2 continues to remain a largely invisible disease even though it kills more Americans and costs more to treat than cancer.

Only a few attempts have been made to stage diabetes and these approaches are not easy to use and have not been widely accepted.^5^–^7^ Gibson et al used macrovascular and microvascular complications to categorize DM2 into four stages with more severe macrovascular complications resulting in higher DM2 stage^3^,^4^ but the distinction between macrovascular complications seemed arbitrary at times.^5^ For example, coronary artery bypass graft resulted in DM2 stage 3 designation while a myocardial infarction (MI) resulted in DM2 stage 4 designation even though both events are equally serious manifestations of cardiovascular (CV) disease.^5^ Furthermore, some microvascular complications such as painful diabetic neuropathy, proliferative retinopathy and a history of osteomyelitis and/or bacterial skin infections of the foot led to DM2 stage 4 similar to a patient with an MI but the rationale for this was not clear.^5^ Nonetheless, they did demonstrate higher mortality and costs with higher DM2 stage but the overall sample size was small (n = 379), and the aboriginal ethnicity of the study population limits generalizability.^5^ Garcia de Alba et al tested the feasibility of another DM2 staging called the USEISS DM2 scale using a retrospective cohort of 2,702 Mexican test subjects matched to 400 cross-sectional controls.^6^ USEISS DM2 stage ranged from 1 to 5 and incorporated primary, secondary, and tertiary prevention strategies to treat lower DM2 stages,^1^–^4^ while diabetic complications were only considered at DM2 stage 5.^6^ They also used 32 distinct risk factors to stage and classify “risk control” in persons with and without diabetes and emphasized behavioral counseling across each DM2 stage which raises significant concern about how feasible this system would be in real-world clinic setting where 30–40 minutes are allotted for each new type 2 diabetic patient.^6^ Finally, Wu published a brief note outlining his concept for a DM2 staging system with five different DM2 stages.^7^ Stages 1–2 did not include diabetes complications, while stages 3–5 did take diabetes complications into account but microvascular and macrovascular complications for each stage were not clearly defined and there was no statistical validation provided.^7^ Finally, White’s classification system is one of the earliest attempts to categorize diabetes but it was limited to pregnant woman with diabetes.^8^ In summary, prior DM2 staging attempts are admirable but limited in their clinical impact because they are not easy to use and lack robust statistical validation and generalizability due to ethnic differences.

### The Diabetes Staging System (DSS)

We propose the DSS for DM2 that utilizes macrovascular events, microvascular complications, estimated glomerular filtration rate (GFR), and hemoglobin A1C to stage DM2. Our goal in proposing this staging system is to start a dialogue between various diabetes stakeholders that leads to further improvement and refinement of DSS. We also have an active Institutional Review Board (Brody School of Medicine at East Carolina University, Greenville, NC, USA) research protocol and are in the process of analyzing the data to see if it can provide preliminary validation for DSS.

#### Staging system overview

**Figure 1:**
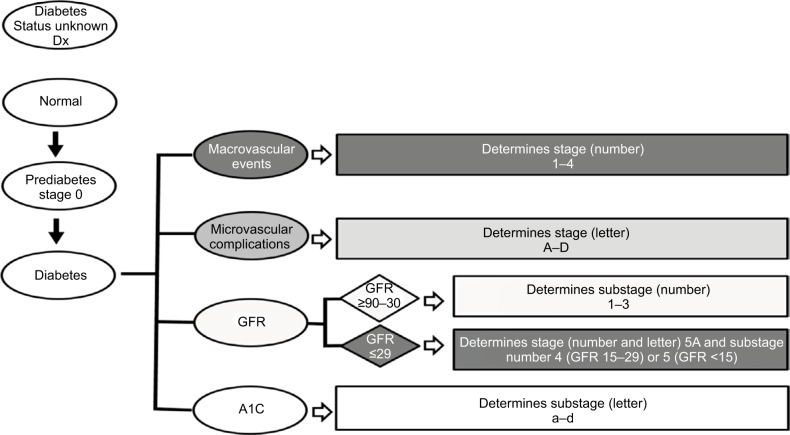
Diabetes Staging System flow diagram for determining stage and substage. **Abbreviation:** GFR, glomerular filtration rate.

**Figure 2:**
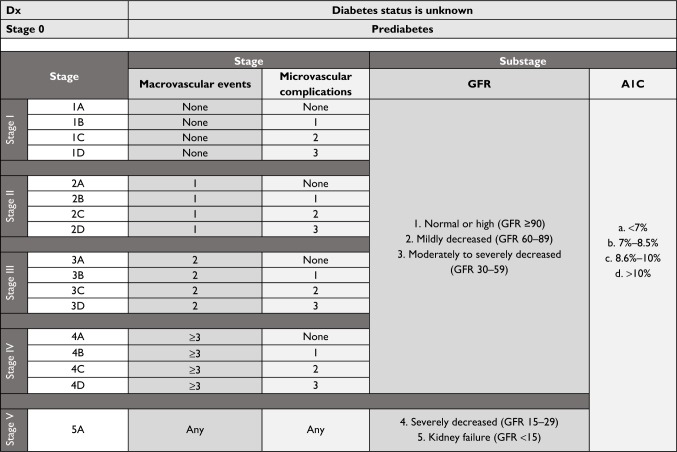
Diabetes Staging System overview. **Abbreviation:** GFR, glomerular filtration rate.

We modeled DSS (Figure 1) on TNM cancer staging because it is well validated and widely accepted. Figure 1 helps the reader visualize how stage and substage are determined. Cancer staging uses the size and extent of tumor invasion to determine disease stage and risk for death. Using this as a paradigm for diabetes staging, we sought to identify a parallel aspect of DM2 that progresses over time and increases mortality and settled on vascular disease. In DSS, macrovascular events are the primary driver of stage with higher stage conferring higher mortality. Microvascular complications reflect morbidity within each stage with more microvascular complications indicating worsening morbidity. In DSS, GFR and A1C are the primary drivers of substage that guides therapy and minimizes harm. Figure 2 shows how DSS would be used in clinical practice. The rationale for DSS is to incorporate well-validated and widely accepted approaches into a broader system that can stage DM2.

#### Macrovascular events determine the stage within DSS

Specifically, the number of macrovascular events or the presence of advanced chronic kidney disease (CKD) defined as Kidney Disease: Improving Global Outcomes (KDIGO) stage 4 (GFR 15–29) or stage 5 (GFR < 15) are the main drivers of a higher diabetes stage.^9^ DM2 stage progresses (Figure 2) with number of macrovascular events as follows: Stage 1 (no macrovascular events) → Stage 2 (one macrovascular event) → Stage 3 (two macrovascular events) → Stage 4 (three macrovascular events). Macrovascular events include MI, coronary revascularization (coronary artery bypass graft), coronary stenting, abdominal aortic aneurysm repair, stroke, transient ischemic attack, peripheral vascular disease requiring revascularization, and amputation of limb including below-knee amputation and above-knee amputation. Stage 5 is the highest stage and is reserved for patients with DM2 who have KDIGO stage 4 or 5 disease regardless of macrovascular events since advanced CKD itself confers a very high risk of death from a macrovascular event. The evidence to justify this approach comes from several large studies including two prospective studies, one pooled analysis, and a National Diabetes Swedish Registry study evaluating cause of death in diabetes.^10^–^13^ These studies found renal disease and then CV disease to have the highest risk for death in diabetics, respectively.^10^–^13^

#### Microvascular complications determine the severity within each stage of diabetes

After DM2 stage is assigned, a numeric value between 1 and 5, an alphabetic uppercase letter between A and D is assigned based on the number of microvascular complications (A = no microvascular complication, B = one microvascular complication, C = two microvascular complications, and D = three microvascular complications). Retinopathy is confirmed by a dilated eye exam, retinal photocoagulation (RP), vascular endothelial-growth factor inhibitor (VEGF-i) injection, vitreous hemorrhage, macular edema, and legal blindness. Nephropathy is confirmed by persistently elevated urine microalbumin/creatinine ratio and decreased GFR. Neuropathy is confirmed by a detailed foot exam that detects the presence of decreased sensation to light touch, pin prick, proprioception, vibration; a history of foot ulcer, amputation of toes/foot/leg, neuropathic ulcer, or Charcot foot. The evidence to justify the use of microvascular involvement as a marker of morbidity comes from several studies that show microvascular complications decrease health-related quality of life.^14^–^17^

#### Glomerular filtration and hemoglobin A1C determine the substage

The nomenclature for diabetes stage (numeric 1–5 and alphabetic uppercase A–D) is followed by nomenclature for substage (numeric 1–5 and alphabetic lowercase a–d). The first part of the substage comprises renal function (number) and the second part (lowercase letter) glycemic control (Figure 2). Renal function is assigned a substage numeric value between 1 and 5 (1- GFR ≥90, 2- GFR 60–89, 3- GFR 30–59, 4- GFR 15–29, 5-GFR < 15), which corresponds to the KDIGO system for classifying kidney function.^9^ As mentioned previously and seen in Figure 2, the only exception is when GFR is 15–29 or GFR < 15; in this case, the severe CKD also determines the stage number (5) and letter (A). The intent of the renal function (GFR) component is to guide therapy in the setting of advanced CKD (GFR < 30) by limiting patient exposure to oral and injectable agents contraindicated in advanced renal disease (ie, Metformin, Sulfonylurea, SGLT-2, TZD, and GLP-1) and to minimize risk for hypoglycemia (ie, decrease insu lin), heart failure (ie, decrease dose of dipeptidyl-transferase 4 inhibitor), and lactic acidosis (ie, decrease or stop Metformin).

#### Renal dosing Recommendations

When the GFR drops to <30, the provider needs to reevaluate the patient diabetes regimen and some changes may be indicated depending on the medication. For example, no dose adjustment is needed for thiazolidinediones, bile acid sequestrants, Dopamine 2-agnoists and Amylin.^34^ Dose adjustment is needed for Repaglinide/Nateglinide, Glipizide/Amaryl, DPP4-inhibitors and insulin.^34^ However, discontinuation is needed for Metformin, Alpha-glucosidase inhibitors, Glyburide, Glucagon-like-peptide I and SGLT-2 inhibitors.^34^

Glycemic control measured by A1C comprises the second part of the substage and is assigned a lowercase letter (a: <7%, b: 7%–8.5%, c: 8.6%–10%, d: >10%). In the event that A1C is confounded by conditions that affect red blood cell turnover (ie, hemolytic anemia, recent blood transfusion, drugs that stimulate erythropoiesis, end-stage kidney disease, and pregnancy), a calculator (http://professional.diabetes.org/eAG) that converts A1C to estimated average glucose (eAG) as follows will be used: <7% → <154 mg/dL, 7%–8.5% → 154–200 gm/dL, 8.6%–10% → 200–240 mg/dL, >10% →>240 mg/dL. Since most blood glucose monitors provide 7-day, 14-day, and 30-day blood glucose average, we will use the 30-day blood glucose average as a surrogate for A1C to determine the second part of the substage. Glycemic control has been shown to predict likelihood to develop microvascular complications in DM2,^18^ and its intent is to help overcome clinical inertia to treatment intensification when glycemic goals are not being met.

### Significance

DM2 is a pervasive and costly condition to treat like cancer. While there are stark differences in the two conditions, both involve gradual progression of a disease and the availability of various treatment modalities. Given the amount of information a provider has to deal with when managing a patient with diabetes, we wanted to create a simplified system of classification that can do for diabetes what staging has done for cancer. We especially liked the straightforward alphanumeric TNM cancer classification given its ability to stage cancer, predict mortality, and guide treatment. In so doing, a complex condition is simplified in a way that allows patients and providers to understand what is at stake and what can be done.

We acknowledge that unlike cancer, diabetes does not have a similar linear progression so it is difficult to stage in a similar way. The other important point to remember with DSS stage is that it does not move backward to a lower stage, it can only progress to a higher stage. A higher DSS stage is considered if additional macrovascular or microvascular complications have developed over the past year. On the other hand, the substage is more variable since it is determined by GFR and A1C which may change based on several factors. Nonetheless, DSS still provides a simplified communication mechanism between providers with easy-to-follow categorization that can be useful in many scenarios, some of which have been highlighted.

#### Improved ability to predict survival

**Figure 3:**
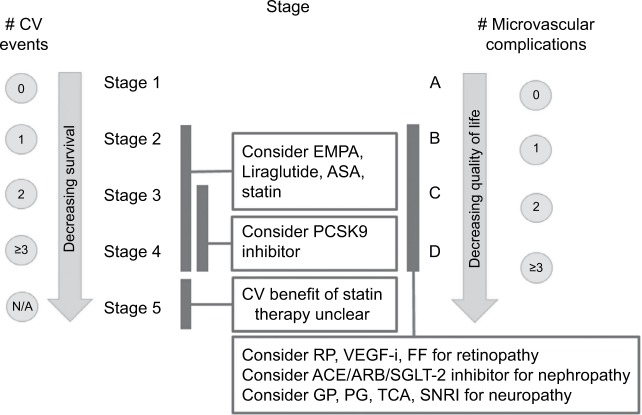
Diabetes Staging System stage based interventions. **Abbreviations:** ACE, angiotensin converting enzyme inhibitor; ARB, angiotensin receptor blocker; ASA, aspirin; CV, cardiovascular; DSS, Diabetes Staging System; EMPA, empagliflozin; FF, fenofibrate; GP, gabapentin; PCSK9, proprotein convertase subtilisin/kexin type 9; PG, pregabalin; RP, retinal photocoagulation; SNRI, serotonin norepinephrine reuptake inhibitor; TCA, tricylic antidepressant; VEGF-i, vascular endothelial growth factor inhibitor.

DSS would allow patients and providers to have a more meaningful discussion about likelihood of survival that has not previously been available. The absence of this information has in our view undermined efforts to encourage lifestyle changes that promote better diet and exercise. In contrast, TNM cancer staging’s ability to quantify mortality has helped providers and patients focus on interventions that help them live longer. The focus on helping DM2 patients live longer is particularly relevant now because recently completed clinical trials such as LEADER and EMPA-REG have for the first time shown that Liraglutide and Empagliflozin prolong life in DM2 patients with established CV disease.^19^,^20^ Since DSS incorporates macrovascular events, it could help guide providers to preferentially prescribe Liraglutide and/or Empagliflozin in DM2 patients with known CV disease (Figure 3) with established CV disease, which would be DSS stages 2–4.^19^,^20^ Furthermore, DSS would be flexible enough to allow other agents demonstrating a mortality benefit in DM2 patients to be added in the future so that this system would adapt to new emerging therapies.

#### Improved ability to guide therapy toward best practices for DM2

**Figure 4:**
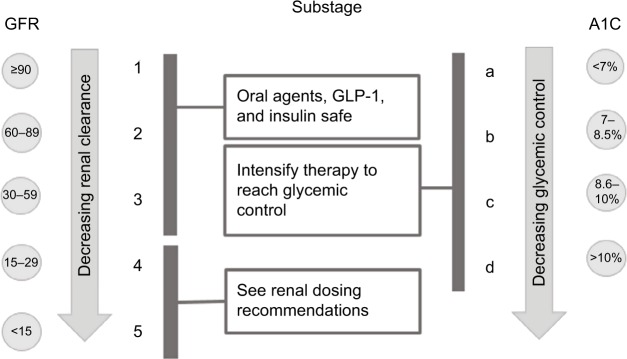
Diabetes Staging System substage based interventions. **Abbreviations:** DPP-IV, dipeptidyl peptidase four inhibitors; DSS, Diabetes Staging System; GFR, glomerular filtration rate; GLP-1, glucagon-like peptide 1.

Primary care providers, clinical pharmacists, and endocrinologists represent the first and often only line of defense for many patients with DM2. During a 30–40-minute visit, they must somehow convey the seriousness of the disease while navigating the myriad 30+ treatment options to find the safest and most effective regimen that will help improve DM2 outcomes. Too many choices sometimes make it difficult to decide which agent to use. Although a formulary system helps reduce treatment options, providers are still left with many choices. The DSS could help in this regard by highlighting evidence-based interventions that have been shown to help improve macrovascular and microvascular complications while also avoiding certain agents that may cause harm. Figures 3 and 4 provide a practical example of an evidence-based “cheat sheet”, which could be made available to providers to reflect the current “state of the art” for DM2 care.

#### PCSK9 inhibitors

DM2 patients with DSS stages 2–4 disease who are continuing to have CV events despite high-intensity statins could also be candidates for further low-density lipoproteins (LDL) lowering using the newer PCSK9 inhibitors which have demonstrated robust LDL lowering below 70 mg/dL and decreased CV mortality in high-risk patients.^21^,^22^ DSS could help health care systems and its providers make more rational treatment decisions by only selecting patients who have failed proven therapies (ie, aspirin, statin, Empagliflozin, and Liraglutide) that are more affordable.

### Treating diabetic retinopathy, nephropathy, and neuropathy

Several studies have shown benefit of RP, VEGF-I, and Fenofibrate^23^–^25^ in treating diabetic retinopathy. With regard to diabetic nephropathy, large clinical trials have shown that angiotensin-converting enzyme inhibitors and angiotensin receptor blockers are effective in preventing onset and progression of nephropathy.^26^,^27^ Gabapentin, pregabalin, tricyclic antidepressants, and serotonin–Norepinephrine Reuptake Inhibitors have been shown to be effective in improving neuropathic pain.^28^–^31^ Figures 3 and 4 provide a summary of the key items discussed above that would be incorporated into DSS and could be developed into a best practice “cheat sheet” that providers could use so that beneficial interventions are not overlooked and patient harm is avoided.

#### Improved ability to predict cost

DSS may also allow health care systems to better predict DM2-related costs since it takes the number and severity of DM2-related macrovascular/microvascular complications into account. For example, DSS could help refine Health Data Information Set (HEDIS), which is currently used by most private health care plans to measure “quality of care” for chronic conditions such as DM2. All type 2 diabetics are currently treated the same by HEDIS but DSS could be used to more accurately reflect severity of macrovascular/microvascular complications in DM2 patients, which would help create better “buy in” from providers and health care systems because they would know that disease acuity is being considered rather than a “one size fits all” approach that shifts all the risk of severe DM2 care on them.

#### Public awareness

DSS can also be used to increase patient and public awareness about the seriousness of DM2 like TNM has done for cancer. Unfortunately, diabetes suffers from a public perception that it is a chronic disease much like hypertension that does not really kill its victim or progress to any real extent. The public has a very visceral “fight or flight” response when the word cancer is spoken. Central to this narrative is the ability of cancer staging to quantify in simple terms the likelihood of death. Since the public is already aware of cancer staging, using a similar approach for DM2 could help change the public’s perception of DM2. Telling someone to change their diet and exercise because they have DM2 is one thing, but quantifying their risk of death may more strongly motivate the patient to change their diet and exercise behavior.

#### Clinical trials

Pharmaceutical companies are always seeking more efficient ways to conduct clinical trials. One core element in conducting a clinical trial is recruiting the right patient population. DSS could make it easier for pharma companies to recruit patients for large multi-center studies by creating a single line statement that reflects CV disease, microvascular complications, GFR, and current glycemic control. This would improve communication between key stakeholder such as pharma companies, the FDA, physicians, and patients. Since the FDA is currently requiring all novel agents for DM2 to demonstrate CV safety, a hypothetical pharma trial could use the DSS to target recruitment of diabetic patients with a stage 2–4 disease and to exclude stage 1 and 5 patients due to low likelihood of CV events or very high CV risk, respectively. Stage 5A patients may also be excluded or require additional monitoring due to their higher vascular risk and inability to clear drugs that are cleared by the kidney. Finally, the DSS could also help pharmaceutical companies to more easily define the patient population best suited for treatment of effective but very expensive agents such as PCSK9 inhibitors.

## Conclusion

Gerber et al noted that in learning machines and humans, the relationship between information load and information handling takes the shape of an inverted U.^32^ As information load increases, so does the ability to handle information but only up to a certain point after which the ability to handle information declines and errors increase. We believe this is one of the factors at play in the clinical management of DM2 that leads to inertia. In fact, clinical inertia is overwhelmingly the major reason that patients remain poorly controlled for years.^33^ We feel that inertia is occurring in part because of information overload especially in primary care practices. Providers often have to deal with multiple chronic diseases during a single visit. For example, there are 34 agents for DM2 and the 2018 ADA standards of care is 153 pages long. How can a primary care provider keep up with such information? DSS can help by distilling the best available scientific evidence into a logical framework that is easy to follow. Specifically, DSS can help clinical inertia in the following three important ways: 1) Improving understanding by streamlining communication between providers and with patients for better coordination and management of diabetes; 2) Increasing survival by emphasizing the importance of CV disease and steering providers toward agents proven to lower CV mortality (ie, Empagliflozin, Liraglutide); and 3) Avoiding harm by including an expansive list of diabetic medications (Figure 4) and recommended actions when GFR falls to <30, which will help the provider make an informed decision that balances risk vs benefit.

To our knowledge, TNM cancer staging has not been previously used as a template for a DM2 staging system. We acknowledge that DSS needs to be validated and are actively working to accomplish this important next step. We also are aware that DM2 patients with a lower DSS stage may not get as much clinical attention as those with established CV events. However, we would like to emphasize that we strongly believe that DSS should be evidence based so if new data emerge demonstrating a particular diabetic medicine to be effective for primary prevention of CV disease then we would be the first to incorporate it into DSS. We also accept that diet and lifestyle play a very important role in management of DM2 and that changing human behavior is one of the most difficult things to accomplish in medicine. However, we feel that the DSS can help change patient perception of their diabetes from a “nebulous” condition to one where they can “see” severity and progression through a simple, step-wise staging system which could motivate them to become more engaged in improving their diabetes through lifestyle, diet, and exercise.

This special article shares our vision for DSS and seeks constructive criticism, feedback, and suggestions from colleagues in the field on how to improve on it. In so doing, we hope that a staging system can be refined over time that is widely adopted by all stakeholders impacted by the disease and that it can make a meaningful impact in improving care and outcomes for DM2, and thereby improving the lives of our patients who have diabetes.

### Case study

A 53-year-old white male with a past medical history of hypertension, hyperlipidemia, obesity, coronary artery disease requiring cardiac stenting, and DM2 presents to our clinic. The patient has had DM2 for the past 10 years. He has had one macrovascular complication namely cardiac ischemia requiring stenting. He is also noted to have diabetic nephropathy and retinopathy based on an elevated urine microalbumin/creatinine ratio and recent dilated eye exam, respectively. Physical examination is positive for decreased sensation to a 5.07 Semmes-Weinstein monofilament and decreased vibratory and position sense in both feet. Sensation to pinprick is normal. Lab results show the following: hemoglobin A1C 9%, GFR 55, urine microalbumin/creatinine ratio 120 mg/gCr, and finger stick blood sugar 200 mg/dL. Fasting lipid panel: total cholesterol 180 mg/dL, high-density lipoproteins 45 mg/dL, LDL 99 mg/dL, and triglycerides 150 mg/dL. Current medication: Metformin 1,000 mg twice per day, Amaryl 4 mg twice per day, Aspirin 81 mg qd, and Lipitor 40 mg qd.

#### What is this patient’s stage using DSS?

Stage depends on the number of macrovascular events and microvascular complications (Figure 2). This patient has one macrovascular event (ie, CAD requiring stenting) and three microvascular complications such as diabetic nephropathy (ie, patient has urine screen positive for moderate microalbuminuria), diabetic retinopathy (ie, patient’s dilated eye exam that showed retinopathy), and diabetic neuropathy (physical exam showed decreased sensation to light touch). Therefore, looking at Figure 2 makes patient’s stage 2D.

Substage is listed next to stage and is determined by GFR and hemoglobin A1C. This patient’s GFR is 55 and A1C is 9%. Therefore, looking at Figure 2 this patient’s substage is 3c (Figure 2).

Therefore, this patient’s overall stage is 2D3c.

#### Is there anything more that could be done to lower his CV risk? How could the DSS help in this situation?

Recent randomized clinical trials such as LEADER and EMPA-REG have shown lower mortality vis-à-vis placebo in type 2 diabetic patients with established CV disease who were randomized to Empagliflozin and Liraglutide, respectively.^19^,^20^ Since the DSS accounts for macrovascular events, it could guide clinicians to more systematically consider Liraglutide and/or Empagliflozin in DM2 patients with established CV disease, which according to our staging system would be stages 2–4.^19^,^20^ Since our patient has stage 2 disease and has already had one macrovascular event, he does merit consideration for Liraglutide or Empagliflozin even though he is on an ASPIRIN and STATIN. Stage 5 patients would be contraindicated due to advanced CKD. The primacy of vascular disease in determining DM2 stage makes it possible for other diabetes agents that demonstrate CV mortality reduction to be added to Liraglutide and Empagliflozin.

The patient is concerned about his future risk for a cardiac event since he has already had a CV event. He cites fears that his brother died of an MI in his fifties, and he does not want to be next as he has two young children and a wife. He wonders whether more could be done to lower his CV risk beyond taking Aspirin and Lipitor. He is aware of recent clinical trials of Liraglutide, Empagliflozin and PCSK9 inhibitors that have decreased CV mortality and wonders if he is a candidate for any of these agents.

#### He wonders if he is a candidate for this agent instead. How could the DSS help in this situation?

DM2 patients with DSS stage 2–4 disease who are continuing to have CV events despite high-intensity statins could be candidates for further LDL lowering using the newer PCSK9 inhibitors which have demonstrated robust LDL lowering below 70 mg/dL and were very recently demonstrated to decrease CV mortality in high-risk patients.^21^,^22^ However, there are a couple of reasons why our patient should not be considered at this time for this therapy. First, his Lipitor dose could be increased further from 40 mg to 80 mg qd as tolerated. Second, either Empagliflozin or Liraglutide is being added to further decrease CV risk and sufficient time needs to be given to these agents before consideration of an even more expensive agent. In this case, DSS could help physicians and health insurers make a more rational decision by trying two other proven less costlier options before considering a much more expensive option such as a PCSK9 inhibitor.

## References

[1] Dall TM, Yang W, Halder P (2014). The economic burden of elevated blood glucose levels in 2012: diagnosed and undiagnosed diabetes, gestational diabetes mellitus, and prediabetes. Diabetes Care.

[2] Boyle JP, Thompson TJ, Gregg EW, Barker LE, Williamson DF (2010). Projection of the year 2050 burden of diabetes in the US adult population: dynamic modeling of incidence, mortality, and prediabetes prevalence. Popul Health Metr.

[3] Odicino F, Pecorelli S, Zigliani L, Creasman WT (2008). History of the FIGO cancer staging system. Int J Gynaecol Obstet.

[4] Chen G, McQuade JL, Panka DJ (2016). Clinical, molecular, and immune analysis of dabrafenib-trametinib combination treatment for braf inhibitor-refractory metastatic melanoma: a phase 2 clinical trial. JAMA Oncol.

[5] Gibson OR, Segal L, McDermott RA (2012). A simple diabetes vascular severity staging instrument and its application to a Torres Strai Islander and aboriginal adult cohort in North Australia. BM Health Services Research.

[6] García de Alba JE, Salcedo Rocha AL, Colunga Rodríguez C, González Barrera JA, Herrera Solís E, Milke Najar ME (2005). UISESS scale for staging and classifying clinical-epidemiological risk in type 2 diabetes mellitus and for establishing multidisciplinary preventive actions. Prev Med.

[7] Wu SL (2015). Staging of type 2 diabetes mellitus. Genet Mol Res.

[8] White P (1978). Classification of obstetric diabetes. Am J Obstet Gynecol.

[9] Levey AS, Coresh J, Balk E, National Kidney Foundation (2003). National Kidney Foundation practice guidelines for chronic kidney disease: evaluation, classification, and stratification. Ann Intern Med.

[10] Rao Kondapally Seshasai S, Kaptoge S, Thompson A, Emerging Risk Factors Collaboration (2011). Diabetes mellitus, fasting glucose, and risk of cause-specific death. N Engl J Med.

[11] Alegre-Díaz J, Herrington W, López-Cervantes M (2016). Diabetes and Cause-Specific Mortality in Mexico City. N Engl J Med.

[12] Tancredi M, Rosengren A, Svensson AM (2015). Excess mortality among persons with type 2 diabetes. N Engl J Med.

[13] Baena-Díez JM, Peñafiel J, Subirana I, FRESCO Investigators (2016). Risk of cause-specific death in individuals with diabetes: a competing risks analysis. Diabetes Care.

[14] Quality of life in type 2 diabetic patients is affected by complications but not by intensive policies to improve blood glucose or blood pressure control (UKPDS 37) (1999). U.K. Prospective Diabetes Study Group. Diabetes Care.

[15] Coffey JT, Brandle M, Zhou H (2002). Valuing health-related quality of life in diabetes. Diabetes Care.

[16] Redekop WK, Koopmanschap MA, Stolk RP, Rutten GE, Wolffenbuttel BH, Niessen LW (2002). Health-related quality of life and treatment satisfaction in Dutch patients with type 2 diabetes. Diabetes Care.

[17] Glasgow RE, Ruggiero L, Eakin EG, Dryfoos J, Chobanian L (1997). Quality of life and associated characteristics in a large national sample of adults with diabetes. Diabetes Care.

[18] Effect of intensive blood-glucose control with metformin on complications in overweight patients with type 2 diabetes (UKPDS 34) (1998). UK Prospective Diabetes Study (UKPDS) Group. Lancet.

[19] Zinman B, Wanner C, Lachin JM, EMPA-REG OUTCOME Investigators (2015). Empagliflozin, cardiovascular outcomes, and mortality in type 2 diabetes. N Engl J Med.

[20] Marso SP, Daniels GH, Brown-Frandsen K, LEADER Steering Committee; LEADER Trial Investigators (2016). Liraglutide and cardiovascular outcomes in type 2 diabetes. N Engl J Med.

[21] Ridker PM, Revkin J, Amarenco P, SPIRE Cardiovascular Outcome Investigators (2017). Cardiovascular efficacy and safety of bococizumab in high-risk patients. N Engl J Med.

[22] Sabatine MS, Giugliano RP, Keech AC, FOURIER Steering Committee and Investigators (2017). Evolocumab and clinical outcomes in patients with cardiovascular disease. N Engl J Med.

[23] Patz A, Fire S, Finkelstein D (1978). Photocoagulation treatment of proliferative diabetic retinopathy: the second report of diabetic retinopathy study findings. Ophthalmology.

[24] Nguyen QD, Shah SM, Khwaja AA, READ-2 Study Group (2010). Two-year outcomes of the ranibizumab for edema of the mAcula in diabetes (READ-2) study. Ophthalmology.

[25] Keech AC, Mitchell P, Summanen PA, FIELD study investigators (2007). Effect of fenofibrate on the need for laser treatment for diabetic retinopathy (FIELD study): a randomised controlled trial. Lancet.

[26] Effects of ramipril on cardiovascular and microvascular outcomes in people with diabetes mellitus: results of the HOPE study and MICRO-HOPE substudy (2000). Heart Outcomes Prevention Evaluation Study Investigators. Lancet.

[27] Parving HH, Lehnert H, Bröchner-Mortensen J, Irbesartan in Patients with Type 2 Diabetes and Microalbuminuria Study Group (2001). The effect of irbesartan on the development of diabetic nephropathy in patients with type 2 diabetes. N Engl J Med.

[28] Vinik A, Emir B, Parsons B, Cheung R (2014). Prediction of pregabalin-mediated pain response by severity of sleep disturbance in patients with painful diabetic neuropathy and post-herpetic neuralgia. Pain Med.

[29] Vinik A, Emir B, Cheung R, Whalen E (2013). Relationship between pain relief and improvements in patient function/quality of life in patients with painful diabetic peripheral neuropathy or postherpetic neuralgia treated with pregabalin. Clin Ther.

[30] Max MB, Culnane M, Schafer SC (1987). Amitriptyline relieves diabetic neuropathy pain in patients with normal or depressed mood. Neurology.

[31] Goldstein DJ, Lu Y, Detke MJ, Lee TC, Iyengar S (2005). Duloxetine vs. placebo in patients with painful diabetic neuropathy. Pain.

[32] Garber AJ, Abrahamson MJ, Barzilay JI (2017). Consensus statement by the American association of clinical endocrinologists and American college of endocrinology on the comprehensive type 2 diabetes management algorithm - 2017 executive summary. Endocr Pract.

[33] Pantalone KM, Misra-Hebert AD, Hobbs TM (2018). Clinical inertia in type 2 diabetes management: evidence from a large, real-world data set. Diabetes Care.

[34] American Diabetes Association standards of medical care in diabetes–2018 (2018). Diabetes Care.

